# Metabolism and Aging: New Approaches to Nutrition and Diet in Aging

**DOI:** 10.1093/geroni/igaa057.2621

**Published:** 2020-12-16

**Authors:** Blanka Rogina

## Abstract

Aging is associated with a functional decline in metabolic, physiological, proliferative, and tissue homeostasis leading to deterioration at an organismal level and increases risk for disease and death. Genetic, pharmacological and nutritional interventions have been successfully used to preserve metabolic health, which leads to preserved healthspan and extended longevity. This symposium will discuss new approaches to nutrition and diet and mechanisms underlying interventions such as calorie restriction and genetic CR. We will also discuss species-specific metabolic mechanisms based on longitudinal studies in mice, monkeys and humans.

